# Nutrigenomics and Precision Nutrition in Inflammatory Bowel Disease

**DOI:** 10.3390/genes17070806

**Published:** 2026-07-15

**Authors:** Sara Jarmakiewicz-Czaja, Aneta Sokal-Dembowska, Kacper Helma, Krystian Krysa, Rafał Filip

**Affiliations:** 1 Faculty of Health Sciences and Psychology, Collegium Medicum, University of Rzeszow, 35-959 Rzeszow, Poland; 2 University Center for Research and Development in Health Sciences, University of Rzeszow, 35-959 Rzeszow, Poland; 3 Department of Gastroenterology with IBD Unit, Clinical Hospital, 35-301 Rzeszow, Poland; 4 Faculty of Medicine, University of Rzeszow, 35-959 Rzeszow, Poland

**Keywords:** nutrigenomics, inflammatory bowel disease, Crohn’s disease, ulcerative colitis, dietary components

## Abstract

Inflammatory bowel disease (IBD) represents a growing public health challenge worldwide, and its prevalence is increasing year by year. A growing body of evidence suggests that diet plays a key role in shaping the gut microbiota, whose dysbiosis is a significant component of IBD pathogenesis. In response to the limitations of traditional, one-size-fits-all dietary recommendations, the concept of precision nutrition is emerging, which involves tailoring nutrition to a patient’s clinical, biological, and genetic characteristics. Of particular importance in this context is nutrigenomics, the science that studies the impact of dietary components on gene expression and the modulation of immune processes. Dietary components, such as polyphenols and certain vitamins, may influence gene expression and immune pathways. However, direct evidence that specific nutrients directly regulate genes associated with inflammatory bowel disease in humans remains limited. Nutrigenomic mechanisms reveal complex relationships between diet, gut microbiota, and genetic predispositions in IBD, encompassing transcriptome regulation, metabolomic changes, and epigenetic processes. A personalized nutritional approach, based on regular assessment of clinical and biological parameters and the close integration of dietary therapy with comprehensive treatment, appears to be a promising direction for the further development of IBD therapy.

## 1. Introduction

Inflammatory bowel diseases (IBD) are chronic, recurrent diseases of the gastrointestinal (GI) tract. The main forms of IBD are Crohn’s disease (CD) and ulcerative colitis (UC). In CD, the inflammatory process can involve all layers of the intestinal wall and can affect the entire length of the GI tract, whereas UC typically affects only the mucosal layer of the colon or rectum [1,2].

IBD are a serious public health challenge worldwide, and the incidence of these inflammatory diseases continues to rise. Between 1990 and 2019, the number of cases increased by 47.5% and it is estimated that in 2019, the number of IBD cases reached approximately 4.9 million, posing a significant challenge for gastroenterologists worldwide [3].

In terms of the prevalence of IBD, based on studies published between 2000 and 2022, Europe remains the continent with the highest rates compared to global estimates (348.4 vs. 229.7 per 100,000), which is mainly due to the particularly high prevalence of UC (198.6 vs. 120.4 per 100,000) [4]. Interestingly, Oceania leads in CD prevalence (173.6 vs. 84.2 per 100,000) and exhibits the highest incidence rates for both CD (12.2 vs. 4.0 per 100,000 person-years) and IBD overall (21.3 vs. 9.7 per 100,000 person-years) [4]. The highest incidence rates for UC are reported in North American populations (9.8 vs. 5.0 per 100,000 person-years) [4].

### 1.1. Role of Diet

The link between IBD development and diet has been partially investigated, but the evidence regarding the impact of specific nutrients remains inconclusive. Nevertheless, it appears that overall diet quality is one of the key factors in the development of IBD. A meta-analysis by Li et al., demonstrated that a Western-pattern diet, characterized by low intake of fruits and vegetables and high intake of red and processed meats, animal fats, and refined grains, is associated with an increased risk of all types of IBD (RR 1.92; 95% CI 1.37–2.68) [5]. On the other hand, there is the Mediterranean diet, which is a dietary plan based on high or moderate consumption of vegetables, fruits, whole grains, nuts, and foods rich in polyphenols [6]. It is considered a generally healthy diet model that not only helps prevent IBD but can also improve patients’ quality of life and the course of the disease itself. This diet can significantly reduce overall levels of inflammatory markers [7], as well as improve general health through potential improvements in gut microbiota composition and a reduction in the incidence of cardiometabolic diseases [8]. On the other hand, a significant limitation of the Mediterranean diet is that it is based on standardized dietary recommendations. Individual differences in genetic background, gut microbiome composition, or disease phenotype may contribute to varying responses to the same dietary intervention. This suggests the need for a precise approach to nutrition that takes into account the patient’s individual characteristics when formulating dietary recommendations. Diet plays a significant role in shaping the composition of the gut microbiota, and disruptions in the microbiota are a key factor in the pathogenesis of IBD. Emerging evidence demonstrates that dysbiosis is not only a consequence but also one of direct causes of inflammation. Antibiotic and probiotic therapies have been shown to be effective in inducing remission and fecal microbiota transplantation (FMT) has demonstrated therapeutic efficacy in UC. These findings are also supported by the identification of a specific abnormal bacterial flora pattern characteristic of IBD [9]. When it comes to the course of IBD, disruption of the mucosal epithelial barrier caused by gut dysbiosis promotes the increase in inflammation and the development of cancer [10].

### 1.2. Precision Nutrition Concept

Precision nutrition is a concept that moves away from recommending rigid, universal model diets to patients in favor of tailoring dietary recommendations to each patient’s individual characteristics. These characteristics include, among others, the gut microbiome, genetic profile, and lifestyle factors. Thanks to continuous technological advances, gaining insight into these characteristics is becoming increasingly feasible [11], and the use of this information is increasingly sought after due to growing awareness among health professionals, who recognize the need to apply it, even though they typically lack sufficient knowledge on the subject [12,13].

Precision nutrition inherently has an advantage over universal template diets, especially when a patient exhibits individual characteristics that differ from the population average. One example is the effect of dietary fiber, commonly regarded as a health-promoting component, which can produce different outcomes depending on the patients’ gut microbiota. In a study by Armstrong et al., it was demonstrated that beta-fructans can have a pro-inflammatory effect in patients whose microbiome prevents their effective fermentation [14]. In such cases, a precision nutrition approach helps to avoid unnecessary dietary restrictions, allowing for targeted regulation of microbiome. In this context, it may be beneficial to accurately estimate and control the intake of fiber from selected sources.

In this article, we describe the role of nutrigenomics and precision nutrition in IBD, with a particular focus on the interplay between genetic susceptibility, microbiome-related mechanisms, and dietary factors. Additionally, we summarize the mechanisms by which specific dietary components influence gene expression and immune regulation, and we analyze potential future directions for the development of precision nutrition in IBD. This manuscript is a narrative review based on a literature search performed in PubMed, Scopus including publications available through May 2026.

## 2. The Genetic Basis of IBD

A patient’s genetic profile is a fundamental factor influencing the pathogenesis of IBD. Because nutrigenomics focuses on the interactions between food and gene expression, this section summarizes the main genetic variants associated with IBD, which may influence individual response to nutritional interventions and provide a foundation for precision nutrition. Genome-wide association studies (GWAS) have identified loci associated with IBD risk, which have subsequently enabled the calculation of a polygenic risk score (PRS), that estimates a patients genetic predisposition to a given medical condition by summing the effects of many small genetic variants [15]. In clinical practice, this score has limited utility due to its low precision in the general population. The vast majority of individuals identified as genetically predisposed ultimately do not develop symptoms of the disease, so the utility of PRS is greater in groups of people who have a higher baseline prevalence of IBD [16]. In 2017, Lee et al. demonstrated that the loci that genetically increase the risk of developing CD are distinct from those that influence a patient’s prognosis, which means that the initiation of the disease itself is driven by different genetic mechanisms than its subsequent activity [17]. To put these mechanisms into context, Table 1 provides an overview of the genetic variants that are considered major predisposing or protective factors for IBD, along with their clinical implications.

NOD2 is a gene whose single nucleotide polymorphisms (SNP) were among the first to be linked to susceptibility to CD. The most common of these are SNP8 (R702W), SNP12 (G908R) and SNP13 (L1007fs) [18]. NOD2 itself is a protein that contains a nucleotide-binding domain (NBD), to which two N-terminal CARD domains are attached, followed by 10 tandem leucine-rich repeats (LRRs) located at the C-terminus [19]. NOD2 influences the intestinal immune system by regulating immune responses to the gut microbiota, and its pathogenic variants can lead to an enhanced inflammatory response [20]. The presence of a harmful mutation does not mean that the disease is inevitable; however, it is estimated that between 10 and 27% of patients with CD have NOD2 susceptibility variants, which increase the risk of developing the disease at least threefold [18]. In the case of having two susceptibility variants, the risk may increase up to 40-fold [18].

ATG16L1 is a gene that encodes a protein which is essential at various stages of autophagy. Paneth cells in patients with CD carrying ATG16L1 polymorphisms exhibit granule abnormalities, as well as a reduced number of granules, which may impair the secretion of antimicrobial peptides and consequently contribute to gut microbiota dysbiosis and increased intestinal inflammation [21]. It has been shown that the T300A (rs2241880) variant of this gene is a risk factor for CD and may influence susceptibility to perianal Crohn’s disease (PCD), suggesting that its detection could potentially aid in the early identification of such individuals [22]. In another study by Liu et al. (2018), it was demonstrated that CD patients who carry the rs2241880 variant and smoke tobacco exhibit abnormalities in Paneth cells [23].

IL23R is a gene encoding a subunit of the receptor for cytokine IL-23 [24]. Its rare variants R381Q, G149R and V362I have been identified, which may lead to impaired IL-23R function by reducing its expression on the cell surface, disrupting protein maturation, or decreasing its stability. This results in reduced activation of STAT3- and STAT4-dependent signaling pathways and decreased production of proinflammatory cytokines [25]. Consequently, these mechanisms may support protection against IBD by limiting excessive immune response. IL-23 and IL-12 are key pro-inflammatory cytokines involved in the pathogenesis of IBD, they belong to the same family and share the common p40 subunit, making them a significant therapeutic target for drugs used in IBD [26].

CARD9 is a gene encoding Caspase Recruitment Domain-containing protein 9 (CARD9), an adaptor protein involved in signaling pathways connected to initiating immune and inflammatory responses [27]. *CARD9* S12N variant may lead to impaired activation of the NF-κB, and consequently to increased production of cytokines such as IL-6 or TNFα, a mechanism that is partly responsible for the increased risk of IBD [28,29]. There is also a less common variant—*CARD9* S12NΔ11—which has a protective effect, reducing the risk of developing the disease [28,29].

## 3. Nutrigenomics in IBD

### 3.1. The Concept and Scope of Nutrigenomics in IBD

Nutrigenomics is the field of study concerned with the effects of specific food components on gene expression. Components such as polyphenols and certain vitamins can influence both pro-inflammatory and anti-inflammatory genes, which in turn can modulate the body’s immune response [30]. Nutrigenetics, on the other hand, is a field of study that examines the impact of individual genetic variation on, for example, the absorption, metabolism, and utilization of nutrients, as well as on the organism’s varied response to different dietary factors [31]. In IBD, this is particularly important due to genetic predisposition, combined with overlapping environmental factors that may increase the risk of developing the disease [32,33]. In their paper, Kiani et al. outline the main pillars of nutrigenomics. Some of these can also be applied to patients with IBD, such as malnutrition, which may predispose individuals to genomic instability, genomic diversity among patients from different countries, and, additionally, food choices and availability [34]. Many researchers point out that specific dietary components influence the immune system, the integrity of the intestinal barrier, and the composition of the gut microbiota [35,36]. The effects of such actions are mediated by a variety of molecular mechanisms [11].

### 3.2. Gene–Diet Interactions in IBD

The interactions between genes and specific dietary factors in IBD can be bidirectional. On the one hand, dietary components can influence gene expression, on the other hand, IBD patients with specific genetic variants may respond differently to the same dietary components, which can lead to varying disease outcomes, for example, a correlation has been demonstrated between CD activity, omega-3 intake, and the IL6-174 and TNFα-857 genotypes. Another example is the association between VCR (vitamin D receptor) polymorphisms, vitamin D deficiency, and the risk of CD [37]. With regard to the immune system, diet can regulate the activity of transcription factors, such as NF-κB and PPAR, which play a fundamental role in regulating inflammatory processes in the organism. In their study, Perez-Martinez et al. demonstrated that a high-fat, Western-style diet can cause an increase in NF-κB levels [38]. In addition, NF-κB activation leads to increased expression of pro-inflammatory cytokines such as TNF-α, IL-1β, and IL-6 [39]. PPAR has various isoforms (PPARα, PPARγ, PPARβ/δ), and each of them plays a role in glucose regulation and lipid metabolism [40]. It has also been shown that fatty acids can influence PPAR expression [41]. The available evidence suggests that omega-3 polyunsaturated fatty acids and dietary fiber significantly suppress inflammatory signaling and improve metabolic and immunological outcomes, while NOD2, IL23R, and ATG16L1 are important immunometabolic sensors involved in IBD. However, direct mechanistic and clinical evidence is needed to demonstrate that omega-3 fatty acids or dietary fiber specifically regulate genes associated with IBD [42,43,44]. Nevertheless, genetic variability among patients influences differences in the metabolism of specific dietary components and in the regulation of the organism’s immune response; therefore, patients with IBD may exhibit varied responses to the same dietary interventions [45,46]. Another key factor in gene–diet interactions is the gut microbiota. Specific dietary components influence both the composition and function of gut microbes, which produce metabolites that affect the integrity of the intestinal barrier such as short-chain fatty acids (SCFAs), secondary bile acids, and indole derivatives [47]. In their study, Bai et al. point out that environmental risk factors predisposing individuals to IBD affect people with different genetic variants in various ways, for example, smoking may increase the risk of CD in individuals with the NOD2 cis1007fs polymorphism [37].

### 3.3. Transcriptomics in IBD

Transcriptomic techniques are used to analyze the transcriptome, which is the complete set of RNA molecules present in a cell. Genetic information is encoded in DNA but is expressed through the process of transcription. mRNA acts as a temporary carrier of information, mediating its transmission, while non-coding RNAs perform various additional functions. Transcriptomics allows for the assessment of gene expression levels under specific biological conditions [48]. Transcriptomics has found applications in medicine, including the identification of disease-associated genes, assessment of treatment response, toxicity testing, and other areas [49]. Holgersen et al. report that 92 IBD-associated genes were overexpressed in the inflamed intestinal mucosa. The genes with the highest levels of overexpression common to both conditions (CD and UC) include *REG1A*, *S100A9*, *LCN2*, *CXCL1-2*, and *NOS2* [50]. Other transcriptomic studies have demonstrated significant abnormalities in the expression of genes associated with the immune response, cytokine regulation, and intestinal barrier function, which is particularly relevant in IBD [51,52,53]. The most commonly studied abnormalities involve gene expression related to the immune response and intestinal barrier function, suggesting that changes in these pathways are among the most characteristic transcriptomic features of IBD. Despite their significant importance, their use as diagnostic or prognostic biomarkers in IBD still requires further validation.

In their study, Herrera-Marcos et al. analyzed the effect of diet on transcriptomes in experimental models across various tissues. They observed that monounsaturated fatty acids (MUFAs) can reduce metabolic stress and decrease the expression of pro-inflammatory genes in various tissues [54]. In another study, Mayr et al., using methods such as transcriptomic profiling of intestinal epithelial cells (IECs), demonstrated that ATG16L1 is essential for the transmission of inflammatory stress signals induced by polyunsaturated fatty acids (PUFAs), as well as for the production of arachidonic acid metabolites [55]. Li et al., after conducting a transcriptomic analysis using animal models, identified the *Epha6* and *Muc4* genes as potential mediators of differences in the severity of high-fat diet-induced intestinal inflammation [56]. Dietary interventions can modulate the expression of genes associated with the production of pro-inflammatory cytokines, such as *IL-1β*, *IL-22*, and *IL-6*. Dietary components with anti-inflammatory effects, such as dietary fiber, or more precisely, its metabolites, may be associated with, for example, inhibition of the MAPK signaling pathway and enhanced DNA repair [57]. Current data suggest that diet may influence the expression of genes associated with the inflammatory process. However, this needs to be confirmed in well-designed human studies. Genes involved in inflammation and barrier function are particularly affected; *MUC1* and *MUC4*, which are mucin genes, play a significant role in the pathogenesis of IBD [58]. Furthermore, with specific reference to the intestinal barrier, Wiley et al. observed that chronic stress in rats leads to a reduction in the number of tight junctions in the colonic epithelium, as well as increased chemokine expression, linking stress to a loss of intestinal barrier integrity [59].

### 3.4. Metabolomics in IBD

Metabolomics is the field of science concerned with the study of metabolites (small molecules) in cells, bodily fluids, and other samples. Applications of metabolomics include the study of physiological and pathophysiological processes, as well as the discovery of disease biomarkers [60,61]. In IBD, metabolomics can help distinguish between different phases of the disease (active phase and remission) and assess the response to treatment or aid in the development of new treatments for IBD [62,63,64]. In IBD, significant changes are observed in the profile of metabolites derived from the metabolism of the gut microbiota. Franzosa et al. observed that the metabolomic profile in patients with IBD strongly correlated with the composition of the gut microbiota and the level of inflammation. Metabolites that were elevated in patients with IBD include lactate, primary bile acids, and ceramide [65]. Diet is a key factor shaping the metabolome, as it provides substrates for the metabolic processes of the entire body, as well as for the gut microbiota. As a result, metabolites reflect the complex interactions between diet, gut microbes, and inflammation in the organism [66,67]. SCFAs, particularly butyrate and propionate, promote intestinal homeostasis by modulating gene expression and suppressing pro-inflammatory mediators, including TNF-α and NF-κB signaling [68]. Another example is tryptophan, whose metabolism is disrupted in IBD. Tryptophan metabolites contribute to intestinal homeostasis, and modulation of tryptophan metabolic pathways and related gene expression has emerged as a potential therapeutic target [69,70,71]. Vila et al. note that fecal metabolomic profiling revealed changes in the bile acid profile in patients with IBD, which may have been caused by alterations in the gut microbiota [64]. Therefore, diet may act as a kind of modulator of the gut microbiota and, consequently, of the bile acid profile [72]. There may also be changes in bacterial genes, leading to the activation of BA receptors and the expression of IBD-related genes [73].

### 3.5. Epigenetics in IBD

Epigenetics is the field of science that studies biochemical and structural changes in cells without altering their DNA sequence [74]. The main epigenetic mechanisms include DNA methylation, post-translational modifications of histones, and the regulation of gene expression involving microRNAs [75,76]. DNA methylation is a reversible process that occurs at cytosines in dinucleotide sequences (CpG) through the action of DNA methyltransferases (DNMTs), which transfer a methyl group to form 5-methylcytosine [77]. Post-translational histone modifications, on the other hand, include phosphorylation, methylation, acetylation, ubiquitination, crotonylation, and others. Histone modifications are a key epigenetic mechanism because they regulate chromatin structure and the accessibility of DNA to enzymes [78]. Histone modifications regulated by metabolic pathways may have therapeutic potential in the treatment of diseases [79]. MicroRNAs are small molecules that serve as key regulators of gene expression [80]. The expression and function of microRNAs are regulated at multiple stages of their biogenesis, including during transcription, transport, and stabilization in the cytoplasm. Disruptions in these processes can lead to changes in microRNAs, which affect gene expression regulation and may contribute to the development of various diseases [81]. Epigenetic mechanisms provide a link between environmental exposures and gene expression [82]. Certain dietary components can influence epigenetic mechanisms, leading to changes in gene expression, for example, folates play a key role in DNA methylation processes, while polyphenols and omega-3 fatty acids can modulate histone modifications and microRNA expression, thereby influencing the immune response [83,84]. In IBD, epigenetic changes associated with the immune response, the regulation of epithelial cell apoptosis, and alterations in the permeability of the intestinal mucosal barrier are also observed [85,86]. These changes are modulated by environmental factors, such as diet and smoking, which influence the expression of genes involved in the inflammatory process through epigenetic mechanisms. Consequently, epigenetics serves as a crucial link between genetic susceptibility and environmental influences in the pathogenesis of IBD [87].

Current evidence suggests that specific food components may influence the course of IBD. This may result from the interaction of these components with the gut microbiota. Dietary components affect both the composition and metabolic activity of the gut microbiota, leading to changes in the production of metabolites such as SCFAs, bile acids, and tryptophan metabolites [47]. These, in turn, modulate the immune response, the maintenance of intestinal barrier homeostasis, and gene expression [35,36]. However, further mechanistic and interventional studies are needed to better define the cause-and-effect associations.

Because IBD is a multifactorial disease, a single level of analysis is not sufficient. “Omics” integration allows us to uncover the heterogeneity of IBD [88]. Each of the “omic” levels provides different information, but it is only by integrating them that we can fully understand the complex mechanisms of disease [89].

## 4. Dietary Components Affecting Gene Expression

### 4.1. Polyphenols

To date, over 8000 polyphenolic compounds have been identified, including tannins, saponins, phenolic acids, and flavonoids such as isoflavones, neoflavonoids, and chalcones as well as flavones, flavonols, flavanones, flavanonols, and flavanols. These compounds are present in many foods, such as grains, fruits, vegetables, nuts, spices, and beverages, including tea and alcoholic drinks. They exhibit anti-inflammatory, antioxidant, and anticancer properties. In recent years, attention has also been drawn to their role in modulating the gut microbiota. Both polyphenols and their metabolites can support the growth of beneficial bacteria and inhibit the growth of pathogenic microorganisms [90]. It has been shown that polyphenols can alleviate inflammation by modulating the toll-like receptor 4/nuclear factor kappa-light-chain-enhancer of activated B cells (TLR4/NF-κB), mitogen-activated protein kinase (MAPK), and Janus kinase/signal transducer and activator of transcription (JAK/STAT) signaling pathways. By inhibiting TLR4 receptor activation and blocking NF-κB nuclear translocation, these compounds can lead to a reduction in the production of pro-inflammatory cytokines, such as IL-6, IL-8, IL-1β, IFN-γ (interferon gamma), and TNF-α (tumor necrosis factor alpha), as well as enzymes associated with the inflammatory process, notably iNOS (inducible nitric oxide synthase) and COX-2 (cyclooxygenase-2) [91]. Furthermore, it has been shown that the gut microbiota can increase the bioavailability of polyphenolic compounds [90].

In recent years, researchers have also been exploring the therapeutic potential of polyphenols in the treatment of IBD. At least a dozen compounds have been identified that could help manage the disease. These compounds include, among others, curcumin, resveratrol, quercetin, catechins, chlorogenic acid and silymarin. However, the strength of evidence varies significantly across these compounds. For instance, in vitro cell culture studies have demonstrated that catechins, baicalein, and naringin can modulate gene expression to suppress inflammatory pathways [92]. In animal models of colitis, polyphenols have been shown to maintain epithelial barrier integrity and favorably modulate the gut microbiome [93]. Despite these promising preclinical findings, data from human clinical trials remain limited, with only a few compounds showing reproducible clinical efficacy in pilot patient studies.

#### 4.1.1. Polyphenolic Compounds in the Mediterranean Diet

MED is considered a dietary pattern with strong anti-inflammatory properties, which may be associated with a reduced risk of developing IBD [94]. It is also a dietary plan recommended for patients being treated for IBD [4]. The use of the MED diet by patients with IBD may be associated with improvements in anthropometric parameters, fatty liver, disease activity, and quality of life [95]. However, some analyses suggest that it is difficult to draw definitive conclusions from the available clinical studies due to their heterogeneity. The authors of these studies emphasize the need for high-quality randomized trials to evaluate the long-term use of the MED diet in IBD [96,97]. Although the beneficial effects of polyphenols in the treatment of IBD have been widely documented in the literature, most of the data still come from preclinical studies [93].

Researchers are particularly interested in the components of the Mediterranean diet, including polyphenols. A randomized controlled trial (RCT) conducted by Gualtieri et al. demonstrated that apple-bergamot juice (MAB) is characterized by a high content of polyphenols, including chlorogenic acid, procyanidin B2, epicatechin, and 4-p-coumaroylquinic acid. The administration of MAB juice in combination with the MED diet led to an increase in the expression of antioxidant and immunoregulatory genes, such as *superoxide dismutase 1* (*SOD1*), *macrophage migration inhibitory factor* (*MIF*), *peroxisome proliferator-activated receptor γ* (*PPARγ*), and *vitamin D receptor* (*VDR*) [98]. However, this was a small clinical study with a limited sample size (*n* = 30), and these findings have not yet been replicated in independent cohorts. Changes within the VDR gene have been linked to the development of IBD, and its overexpression in the intestinal epithelium has a protective effect against colitis by regulating the expression of the protein claudin-15 (CLDN15) [99]. Furthermore, antioxidant enzymes such as SOD1, acting in concert with catalase (CAT) and glutathione peroxidase (GPx1), protect against the harmful effects of reactive oxygen and nitrogen species (ROS/RNS). It has been shown that in IBD patients in remission, *SOD1* activity is lower than in healthy individuals, and the presence of the *SOD1 +35A/C* polymorphism (A/C genotype or C allele) is associated with a significantly lower risk of developing IBD, suggesting its protective role [100].

However, despite these promising mechanistic insights, the clinical evidence remains constrained by several limitations. Many available studies, including the trial on MAB juice, often feature small sample sizes and short intervention periods. Therefore, while MED diet polyphenols show strong therapeutic potential, large-scale, well-powered RCTs are still required to confirm their definitive clinical efficacy and to establish standardized dietary protocols for IBD patients.

#### 4.1.2. The Effects of Selected Polyphenols with Therapeutic Potential in IBD

Resveratrol

Resveratrol (RSV) may help combat inflammation in IBD by targeting key signaling pathways including NF-κB, mTOR, SIRT1, and Nrf2 [101]. In preclinical rodent models, (TNBS/DSS-induced colitis), RSV administration at doses of ≥80 mg/kg body weight significantly reduced pro-inflammatory cytokines (TNF-α, IL-1β, IFN-γ) and suppressed the expression of pro-inflammatory genes (iNOS, COX-2, IL-6) [102]. Furthermore, in these animal models, RSV alleviated mucosal damage by upregulating tight junction proteins (ZO-1, occludin, claudin-1) and restoring mucin-2 (MUC2) levels [102,103,104,105]. As is well known, MUC 2 is essential for maintaining the integrity of the mucosal barrier; therefore, impaired production of MUC 2 increases susceptibility to the development of chronic mucosal inflammation [106]. Furthermore, positive shifts in the gut microbiota composition and even reductions in colitis-related neuroinflammation have been observed in animal studies [107]. Despite these hypothetical mechanisms identified in rodent and cell models, robust human clinical evidence evaluating the effect of resveratrol on human IBD is still insufficient, and its definitive therapeutic efficacy has not yet been confirmed.

Curcumin

Clinical data that curcumin supplementation reduced the expression of pro-inflammatory cytokines such as IL-6, IL-1β, IL-12 and TNF-α, while increasing the level of anti-inflammatory cytokines, including IL-10 and transforming growth factor β (TGF-β) [108]. In parallel, preclinical cell-line models indicate its ability to inhibit the activity of inflammatory enzymes such as COX-2 and lipoxygenase, which also appears to be significant [109]. As has been observed clinically in patients with IBD, there is increased lipid peroxidation, particularly during disease flare-ups, which can lead to oxidative damage to the intestinal epithelium [110]. In addition, curcumin may help maintain the integrity of the intestinal barrier by increasing the expression of tight junction proteins [108,111]. In vitro studies further demonstrate its ability to dissociate the Keap1 protein from the Nrf2 transcription factor and to increase its activity and expression. In turn, the activation of Nrf2 leads to the neutralization of ROS/RNS. Furthermore, curcumin may contribute to the increased activity of antioxidant enzymes, such as SOD, in patients with IBD [108]. Elevated activity of both SOD and CAT may increase the expression of TJPs (ZO-1, claudin-1, and occludin), as has been demonstrated in preclinical cell-line models [112].

In terms of clinical evidence, curcumin supplementation in both pediatric and adult populations has been shown to have beneficial effects on the control of IBD symptoms, and in patients with mild flare-ups, it may contribute to achieving remission more quickly. Its mechanism of action is complex and includes the previously mentioned effect of inhibiting the expression of pro-inflammatory genes, including the suppression of NF-κB pathway activation and reduced IL-8 expression. Furthermore, curcumin may inhibit inflammatory responses mediated by NOD2, a gene whose mutations are associated with the development of Crohn’s disease, and modulate the mTOR pathway [113]. The results of randomized controlled trials (RCTs) indicate that curcumin has a beneficial effect on achieving clinical remission, reducing SCCAI scores (a measure of clinical activity), promoting the healing of perianal lesions, and lowering inflammatory markers. Furthermore, its use appears to be safe and is associated mainly with mild side effects, such as bloating, nausea, heartburn, or changes in bowel frequency [108].

According to Shahinfar et al., curcumin supplementation may offer benefits not only in terms of clinical and endoscopic improvement, but also in terms of patients’ quality of life [114]. However, as Goulart et al. point out, assessing the efficacy of curcumin is complicated by the heterogeneity of available RCTs in terms of administration form, dosage, treatment duration, and sample size. When using nanomicelles, therapeutic doses may be lower, ranging from approximately 100–240 mg/day, whereas with standard capsules, they range from 1500–3000 mg/day [108].

### 4.2. Omega-3 Fatty Acids

According to the European Society for Clinical Nutrition and Metabolism (ESPEN), a diet rich in omega-3 fatty acids is associated with a reduced risk of developing IBD [46]. High total fat intake may be slightly associated with the risk of developing IBD, while omega-3 intake has been linked to a protective effect [115]. Omega-3 intake may potentially reduce the risk of IBD recurrence and disease progression, although, as Ajabnoor et al. point out, the quality of the data is rather low, and the study results are conflicting. In human studies, omega-3 doses ranged from 1.12 to 4.5 g of EPA/day plus 0.73 to 2.4 g of DHA/day, and the duration of intervention ranged from 6 to 24 months. Omega-3 supplementation reduced the erythrocyte sedimentation rate (ESR) and levels of C-reactive protein (CRP), but increased calprotectin levels in stool samples. The authors suggest that omega-3 fatty acids may slightly reduce the risk of relapse in inflammatory bowel disease, but do not appear to have a clear anti-inflammatory effect. However, they note that it is essential to design and conduct high-quality studies in this area [116].

Scaioli et al. observed in a small clinical study (*n* = 60) that a dose of 500 mg of EPA twice daily in patients with UC for 6 months may be effective in reducing fecal calprotectin levels by 100 points. Additionally, clinical remission was maintained in over 50% of patients. However, the small sample size and the duration of the study constituted a significant limitation of this analysis. However, this study was limited by its small sample size and short duration [117]. A large-scale systematic review and meta-analysis by Mozaffari et al., which included a total sample size of 282,610 participants, demonstrated a significant inverse association between fish consumption and the risk of CD. To explain these clinical observations, the authors highlighted supporting laboratory data from preclinical models, pointing to the potential role of omega-3 fatty acids in inhibiting the activation of TLR-4, NF-κB, and the PPAR-γ receptor, which underscores their influence on the expression of genes responsible for inflammation [118]. This discrepancy suggests that while omega-3 fatty acids show strong preventive and mechanistic potential, their actual therapeutic efficacy in active IBD remains inconclusive due to highly heterogeneous clinical trial designs.

### 4.3. Fiber

ESPEN highlights the potential role of dietary fiber in maintaining IBD remission, although it does not recommend increasing its intake for this purpose [46]. Given the heterogeneity of the variables in currently available studies, it is difficult to unequivocally determine the effect of dietary fiber on the risk of relapse and disease activity. However, it is believed that dietary fiber intake appears to be safe [119,120]. As is well known, a diet rich in dietary fiber has a beneficial effect on the composition of the gut microbiota and stimulates gut bacteria to produce SCFAs, including butyrate, which has anti-inflammatory properties. However, our understanding of the exact molecular pathways rests largely on animal and cell models. The mechanism by which SCFAs affect the intestinal epithelium is multifaceted. By stimulating anti-inflammatory pathways, dietary fiber helps maintain the intestinal epithelial barrier [42]. In vitro studies show that SCFAs also exhibit antioxidant activity by inhibiting NF-κB and IFN-γ and increasing IL-10 levels [121]. In these cell models, it has been demonstrated that SCFAs (acetate, propionate, lactate, and butyrate) have the ability to inhibit the expression of genes associated with inflammation, including TNF-α and NF-κB. Additionally, they may influence the upregulation of tight junction gene expression (*CLDN4*, *OCLN*, *ZO-1*). The anti-inflammatory effect of butyrate in the gut stems primarily from its role as an HDAC inhibitor, which influences gene transcription regulation. At the same time, it has been shown that SCFAs have the ability to modulate NO signaling independently of their ability to inhibit HDAC activity [122].

### 4.4. Vitamin D

Vitamin D is a fat-soluble steroid hormone that exists in two forms: vitamin D2 (ergocalciferol) and vitamin D3 (cholecalciferol) [123]. According to recommendations from ESPEN and AGA (The American Gastroenterological Association), monitoring 25 (OH) vitamin D levels is crucial in both children and adults with IBD [4,46]. Correcting vitamin D deficiency may be associated with a reduction in hospitalizations among this group of patients [124]. The biological effects of vitamin D are primarily mediated by the VDR [125]. It has been observed that in patients with CD, polymorphisms in the *VDR* gene, particularly the *ApaI* polymorphism, influence the disease phenotype, disease progression, and response to treatment [126]. *VDR* is regulated by a number of miRNAs, and its expression is reduced in patients with IBD. One possible cause of this, demonstrated in cell-line models, is the effect of TNF-α, which lowers receptor levels by activating *miRNA-346* [127]. In a single rodent model study, it was also observed that a diet low in vitamin D leads to increased expression of *miR-142-3p* in ileal tissues, resulting in impaired autophagy and accumulation of the autophagy adaptor p62 in Paneth cells. To validate this clinically, parallel analyses of human IBD patients revealed increased ex-pression of miR-142-3p in colon tissues [128].

In patients with IBD, elevated levels of CRP are negatively correlated with serum 25-(OH)-D levels [129]. Adequate vitamin D intake may help lower CRP levels in these patients [130]. Adequate serum vitamin D levels (>30 ng/mL; 75 nmol/L) have an inhibitory effect on the mRNA expression of proinflammatory cytokines such as TNF-α, IL-1α, and IL-6. Furthermore, vitamin D has been shown to reduce oxidative stress by regulating the expression of antioxidant genes. A 2024 meta-analysis of data, including 1209 patients, confirmed that vitamin D supplementation may be beneficial in both preventing flares and maintaining remission in patients with IBD. However, it should be emphasized that the available studies are characterized by high heterogeneity [131].

### 4.5. Amino Acids

Tryptophan is an essential amino acid and must be obtained from food sources, including dairy products, meat, fish, seeds, and nuts. However, in patients with active IBD, its absorption from the gastrointestinal tract may be reduced and its metabolism impaired [70]. Proper tryptophan metabolism is crucial for maintaining immune homeostasis. Chen et al. observed in a single-cell transcriptomic and bioinformatic analysis of human tissue datasets that in patients with UC, the expression levels of the *CTSS*, *S100A11*, and *TUBB* genes are significantly elevated and influence the regulation of tryptophan metabolic pathways [132].

In a study by Antonio et al., conducted in murine and in vitro models, supplementation with *Lacticaseibacillus rhamnosus GG* (LGG) increased levels of the enzyme ASL (arginine succinate lyase) and, consequently, levels of arginine, which are essential for the production of nitric oxide and the maintenance of tight junctions. While reduced ASL expression is observed in human IBD patients leading to barrier dysfunction, the mechanistic claim that the reparative effect of LGG depends on dietary tryptophan rests currently on these specific preclinical system [133]. Similarly, Hara et al. made an interesting observation, demonstrating that a temporary deficiency of methionine, tryptophan, and niacin led to a decrease in the diversity of bacteria belonging to the order *Bacteroidales*. Importantly, a sharp increase in the *Lactobacillus* population was observed after returning to a normal diet. In this animal model, the deficiency of these nutrients resulted in the activation of the anti-inflammatory PPAR signaling pathway and increased expression of antioxidant genes. Reintroduction of these substances led to a sort of “reset” of the gut microbiome and beneficial changes in host gene expression, promoting the restoration of homeostasis [134]. Whether these dietary restrictions and reintroduction strategies yield the same microbiota ‘reset’ in humans remains to be evaluated in clinical trials.

**Figure 1:**
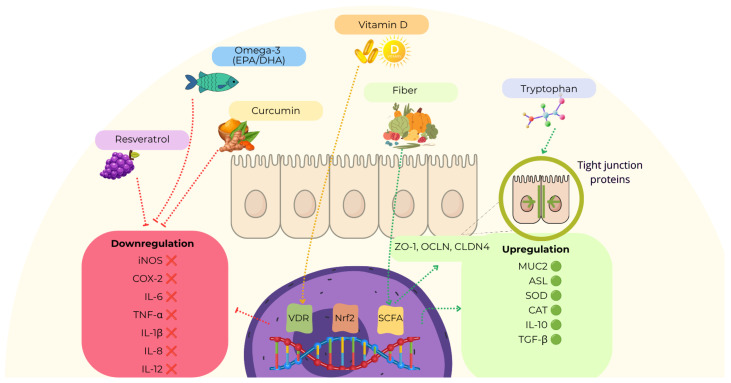
Molecular mechanisms of dietary compounds in maintaining gut barrier integrity and modulating inflammation. Main established pathways of resveratrol, curcumin, omega-3 (EPA/DHA), fiber (via SCFAs), vitamin D, and tryptophan within intestinal epithelial cells. The left panel shows the downregulation of key pro-inflammatory mediators, while the right panel illustrates the upregulation of barrier-protective tight junction proteins, mucins, and antioxidant enzymes. Abbreviations: ASL, argininosuccinate lyase; CAT, catalase; CLDN4, claudin-4; COX-2, cyclooxygenase-2; DHA, docosahexaenoic acid; EPA, eicosapentaenoic acid; IL, interleukin; iNOS, inducible nitric oxide synthase; MUC2, mucin-2; Nrf2, nuclear factor erythroid 2-related factor 2; OCLN, occludin; SCFA, short-chain fatty acids; SOD, superoxide dismutase; TGF-β, transforming growth factor-beta; TNF-α, tumor necrosis factor-alpha; VDR, vitamin D receptor; ZO-1, zonula occludens-1. Source: Figure created by the author based on the above analysis.

Dietary regulation of gene expression is shown in Figure 1.

## 5. Personalized Nutritional Interventions in IBD

Precise nutrition in IBD, is an important element of a comprehensive therapeutic approach in this group of diseases [4,135]. It is based on the assumption that the response to dietary interventions is heterogeneous and depends, among other things, on the disease phenotype [136]. Unlike standard, population-based recommendations—which rely on average effects across entire patient groups and treat patients with the same diagnosis uniformly [137]—the precision approach involves tailoring interventions to the patient’s clinical status [4,138,139], while biomarkers are used to objectively monitor treatment response [140]. Available evidence suggests that nutritional interventions can improve symptom control and nutritional status [4]. However, their impact on maintaining long-term remission remains equivocal [45,141,142].

### 5.1. Personalized Diet Strategies

In clinical practice, planning nutritional interventions takes into account the type of IBD, the location and severity of inflammatory lesions, the disease phase, nutritional status, the presence of complications, and the patient’s dietary preferences and adherence [135,143]. The choice of intervention should therefore be based on the therapeutic goals, available evidence, and the individual circumstances of the patient.

#### 5.1.1. Enteral Nutrition and Exclusion Diets in Crohn’s Disease

Regarding interventions supported by robust randomized controlled trials (RCTs), the strongest evidence currently concerns exclusive enteral nutrition (EEN) in pediatric CD. EEN achieves clinical remission in 60–80% of children with CD and remains the first-line therapy for inducing remission in this age group, regardless of the type of formula used [135,144,145]. The mechanism of action includes reduction in exposure to potentially pro-inflammatory dietary components, modulation of the gut microbiota, and a direct nutritional effect on the mucosa [146,147]. In adults with CD, adherence to EEN is significantly lower than in children, which limits the widespread use of this intervention in practice [143,148]. Partial enteral nutrition (PEN) is an option for patients unable to adhere to EEN, but most studies did not precisely define the composition of solid foods accompanying the formula, which limits the interpretation of results and prevents the formulation of clear recommendations for PEN as a stand-alone strategy [149].

A much better documented elimination strategy is the Crohn’s Disease Exclusion Diet (CDED) [150], designed to be combined with PEN and aimed at limiting gut exposure to nutrients that may disrupt the mucosal barrier and gut microbiota composition, while ensuring proper nutrition [151]. In the study by Levine et al., CDED + PEN did not demonstrate superiority over EEN in inducing corticosteroid-free remission at week 6 in a randomized trial-controlled trial (75% vs. 59%, *p* = 0.38); as the trial was not powered for non- inferiority, this non- significant result indicates a lack of statistically significant difference rather than formal equivalence. However, the CDED + PEN strategy was significantly more effective than a free diet with 25% PEN (not EEN) in maintaining remission at week 12 (75.6% vs. 45.1%, *p* = 0.01). Furthermore, the CDED + PEN approach was associated with significantly better dietary tolerance (97.5% vs. 73.6%, *p* = 0.002) [152]. Preliminary evidence for the efficacy of the CDED in adults was provided by an open-label, pilot randomized trial by Yanai et al., which included biological-naive patients with mild-to-moderate disease. Both CDED combined with PEN and CDED monotherapy were effective in inducing clinical remission at week 6 (68% vs. 57%, respectively; *p* = 0.46); however, the study was underpowered to detect statistically significant differences between the two interventions. Notably, among the subgroup of patients who achieved clinical remission at week 6, 80% maintained it through week 24, which translates to a sustained remission rate of 50% in the overall intention-to-treat (ITT) population. Furthermore, endoscopic remission was confirmed in 35% of the overall ITT cohort at week 24 [153]. It should be noted, however, that aggregate data [45] do not yet confirm durable remission benefit across dietary interventions, and these findings should be interpreted as hypothesis-generating rather than practice-defining.

#### 5.1.2. Symptom-Targeted and Anti-Inflammatory Dietary Patterns

An important area of intervention is modulation of fiber and fermentable carbohydrate (FODMAP) intake. A low-FODMAP diet can reduce functional symptoms such as bloating, pain, and discomfort [154,155,156], but its use is recommended as a strategy to alleviate overlapping IBS- like symptoms rather than as a targeted treatment for active inflammation in IBD [154,155]. Long-term restriction without a reintroduction step can lead to a depletion of gut microbiota diversity, particularly a reduction in the abundance of beneficial taxa, including butyrate-producing ones. *Faecalibacterium prausnitzii* and bacteria of the genus *Bifidobacterium* [157]. Therefore, the decision to limit FODMAPs should be temporary, monitored and aimed at identifying individual tolerance thresholds, with a mandatory reintroduction phase [154,155].

MED and its modifications are most often analyzed as potentially anti-inflammatory patterns [158] in clinical remission [151]; nevertheless, it is important to distinguish that the evidence base for this diet relies primarily on observational studies, alongside a limited number of RCTs. In a prospective observational study, Chicco et al. evaluated 142 IBD patients who followed a MED for 6 months and observed lower rates of active disease and improved quality of life [95]; however, it is important to note that observational designs are susceptible to healthy-adherer bias, which may inflate the apparent benefit. In turn, the study by Lewis et al. showed similar effectiveness of the MED and Specific Carbohydrate Diet (SCD) in terms of reducing clinical symptoms; however, in both groups the response in terms of objective inflammatory markers was relatively low [159]. Importantly, both diets elicited a low response rate in objective inflammatory markers, demonstrating a lack of significant efficacy regarding the key secondary outcomes. Mechanistically, this effect is associated with a greater supply of unsaturated fatty acids, polyphenols, and bacterial fermentation substrates [158].

Among interventions supported primarily by pilot studies, the Ulcerative Colitis Exclusion Diet (UCED) is an emerging strategy worth considering. Although the current data are in the early stages, these studies have demonstrated the potential of this intervention to induce clinical remission in patients with mild to moderate disease. However, due to the still limited high-quality evidence, UCED remains classified as a strategy requiring further evaluation in multicenter studies [160].

#### 5.1.3. Clinical Challenges, Adherence, and Nutritional Deficiencies

Adherence to long-term dietary interventions remains one of the most serious clinical challenges. Barriers to adherence include the cost of specialized products, limited availability of dietary care, cultural differences and dietary habits [138], as well as the psychological burden associated with dietary restrictions in chronic illness [161]. A significant clinical problem remains the phenomenon of independent and often unjustified dietary restrictions undertaken by patients, who often perceive nutrition as a secondary element of standard medical care [162]. Lack of systematic prioritization dietary therapy and education in this area contribute to nutritional gaps and significantly increase the risk of malnutrition [162]. Dietary recommendations alone, without behavioral support and regular dietary supervision, may therefore have limited clinical effectiveness. An additional aspect requiring attention is modifying food texture in patients with intestinal strictures. The American Gastroenterology Association (AGA) recommends cooking, blending, and thoroughly chewing fruits and vegetables in this group, as well as limiting the consumption of sugar-sweetened beverages, which are associated with a more severe course of IBD [4]. Regardless of the chosen nutritional model, the basis of management remains active correction of nutritional deficiencies. Micronutrient deficiencies are common in patients with IBD and primarily involve iron, with iron deficiency anaemia affecting approximately 20% of patients (19.6% in CD and 21.6% in UC) [163]. Monitoring and correcting levels of vitamin D, vitamin B12 (estimated to be deficient in 5.6–38% of the CD population), folic acid, zinc, and copper are also crucial [4]. Regular assessment of nutritional status, supplemented by monitoring laboratory biomarkers, should therefore be an integral part of the care of every IBD patient [4]. Therefore, the role of the dietitian as a nutritional intervention coordinator, patient educator, and adherence monitor is a key, yet often underestimated, element of nutrition.

### 5.2. Biomarkers in Monitoring Nutritional Interventions

Biomarkers are a key element of the treat-to-target strategy for IBD, based on regular monitoring of objective markers of inflammation to optimize therapeutic decisions [164,165]. In the context of nutritional interventions, their role is to assess inflammatory activity, safety, and response to treatment [165,166].

Calprotectin (FC) remains the primary non-invasive marker of intestinal mucosal inflammation [167,168]. FC levels < 50 µg/g are highly sensitive in ruling out active inflammation in the small intestine in CD patients in clinical remission [168], while values < 100 µg/g correlate with deep mucosal remission [169]. However, FC thresholds vary depending on the histological index and analytical kit used, and quantitative agreement between different kits is suboptimal [170]. These factors partially explain the considerable range of reported cut-off values, with histological healing in UC ranging from 40.5 to 250 µg/g [171], which poses a significant challenge in study interpretation and analysis [172]. Furthermore, the time interval between stool sample collection and endoscopy represents an additional source of heterogeneity [170]. All of this requires caution when directly transferring single cutoff values to clinical practice without considering the methodological context of the study. FC cutoff values also differ between CD and UC, as well as depending on the mucosal scoring indices used [173]. Disease location may also influence FC interpretation, which remains a matter of debate in the context of isolated small intestinal lesions in CD [174,175]. These factors further reinforce the need for individual and contextual interpretation of results, demonstrating the limitations of using a single universal cutoff point.

Routine laboratory tests, including C-reactive protein (CRP) and erythrocyte sedimentation rate (ESR), are important but only complementary tools in the diagnosis and monitoring of IBD activity [176]. A significant diagnostic problem is the fact that in approximately 40% of patients with active but mild inflammation, CRP and ESR levels may remain within the normal range, which hinders early detection of exacerbations [176]. Furthermore, the interpretation of these indicators requires caution, as their values may be modified by extraintestinal factors, including concomitant bacterial or viral infections, which reduces their diagnostic specificity [177]. Therefore, an optimal diagnostic model requires the integration of clinical assessment with more sensitive biomarkers [178]. In parallel, nutritional status and micronutrient deficiencies, such as ferritin, should be monitored [179,180]. It should be noted that its concentration increases independently of iron stores in acute and chronic inflammatory states, which may mask the actual deficiency [179,180,181]. It is also worth monitoring the concentrations of vitamin D (25-OH) [179,181], vitamin B12, and folate [180,182]. Metabolite profiles, including short-chain fatty acids (SCFAs), secondary bile acids, and tryptophan metabolites, are also being investigated as potential indicators of biological response to dietary interventions [183]. In a study by Kim et al. [184] advanced serum metabolomic profiling (targeted and untargeted analyses) was used to identify biomarkers that differentiate IBD patients from healthy controls and distinguish between IBD subtypes. Both CD and UC showed elevated levels of tryptophan and indole-3-acetic acid, while the ratio of primary to secondary bile acids decreased. ROC curve analysis confirmed the high diagnostic value of these markers, and the identification of specific changes in tryptophan metabolites allowed for precise differentiation between CD and UC, which could indicate the diagnostic potential of serum metabolomic profiling in personalizing patient care. Despite promising results, microbiome biomarkers and assays are currently not validated for routine clinical use and require further methodological standardization [64,185].

Table 2 summarizes biomarkers used to monitor IBD activity and nutritional interventions.

The most reliable clinical decisions therefore result from integrating multiple layers of data symptoms, biomarkers, endoscopy results, nutritional status, and microbiome data, rather than from interpreting a single parameter. It should be emphasized that most studies assessing these biomarkers are cross-sectional, which prevents causal inference. Prospective interventional studies are required.

### 5.3. Directions for Future Research

Future studies should combine clinical and objective endpoints, including symptomatic remission, mucosal healing, normalization of fecal biomarkers, such as calprotectin [186,187]. Monitoring indicators such as hospitalization rates is also an important complement to assessing the effectiveness of long-term interventions [188]. Multidimensional assessment allows for distinguishing the true anti-inflammatory effect from symptomatic improvement alone [186,189]. The STRIDE-II [187], which precisely defines the timeframe of therapeutic goals, encompassing clinical remission as a short-term goal, normalization of inflammatory biomarkers as an intermediate goal, and endoscopic healing combined with restoration of the patient’s quality of life as long-term goals, could serve as a model for designing such interventions.

Predictive models combining clinical, nutritional, microbiome, and metabolomic data may enable the identification of patients who would most benefit from specific dietary interventions [189,190]. However, the application of these tools in practice requires external validation in independent cohorts and transparent reporting of analytical methods [191,192]. Combining microbiota profiling data with clinical data to predict response to nutritional interventions is under preliminary investigation, but requires further validation before clinical implementation [147,193]. Interventions targeting microbiota modulation are also being intensively investigated [194,195], but the level of evidence remains inconclusive [194,196].

Personalization of nutrition in IBD faces significant obstacles. First, there is limited availability of specialized dietary care. In the 2026 study by the European Society for Paediatric Gastroenterology Hepatology and Nutrition (ESPGHAN) involving 63 physicians from 31 countries, 21% of pediatric IBD centers rarely or never had access to a dietitian, and this access was significantly lower in lower-income countries (31% vs. 66%, *p* = 0.015) [197], while in a UK national survey only 41% of IBD patients reported access to dietary advice when needed [198]. Second, significant organisational differences exist between centres and countries. For example, 45% of respondents in a survey by the European Crohn’s and Colitis Organisation (ECCO) reported not using any nutritional screening tool [199]. It means implementing these dietary therapies would require significant structural changes in gastroenterology practice [151]. Finally, long-term adherence remains a major challenge—a systematic review of 29 studies showed a high prevalence of food avoidance (28–89%) and restrictive dietary behaviors (41–93%) in adults with IBD. These behaviors are frequently driven by dietary misinformation, perceived active disease, and a profound fear of adverse bowel symptoms. While self-imposed restrictions are often intended as a temporary protective mechanism, their unguided prolongation significantly predisposes individuals to nutrition-related complications. Moreover, this chronic restriction carries a substantial psychosocial burden, demonstrably impairing patients’ food-related quality of life [200].

Effective implementation requires multidisciplinary collaboration, with a dietitian as an integral part of the team. IBD teams with a dietitian demonstrate improved clinical outcomes and cost-effectiveness [201]. Supportive tools are also being developed, including mobile apps for tracking symptoms and food intake [202]. Telemedicine solutions have been shown in studies to reduce the number of outpatient visits [203]. However, their impact on long-term clinical outcomes requires further evaluation [204].

## 6. Conclusions

Nutrigenomics mechanisms reveal complex interactions between diet and genetic predisposition in IBD. Through interactions between genes and diet, transcriptome regulation, metabolomic changes, and epigenetic mechanisms, specific dietary components may influence the immune response and the integrity of the intestinal barrier. Understanding these mechanisms forms the basis for the development of personalized dietary strategies to support the treatment of IBD. Particular importance is attached to components such as polyphenols, amino acids, and dietary fiber. A personalization approach based on measurable clinical and biological parameters, regular reassessment of response, and close integration of dietary therapy with a comprehensive IBD treatment plan, while fully utilizing a multidisciplinary team and available technologies, seems to be the most reasonable model for further development in this field. Nevertheless, direct evidence showing that specific dietary components directly regulate genes associated with inflammatory bowel disease in humans remains limited, and further research is needed to confirm these mechanisms.

## Figures and Tables

**Table 1:** Summary of key genetic variants in IBD and its clinical implications.

Gene	Key Variants	Clinical Implications	Function Relevant to IBD Pathogenesis	References
NOD2	- R702W - G908R - L1007fs	- The risk may increase significantly in individuals who carry two variants that increase susceptibility, although the reported magnitude of the effect varies by population. - Found in 10–27% of CD patients.	- Regulation of immune responses to gut microbiota.	[18,19,20]
ATG16L1	- T300A	- Risk factor for CD and PCD. - Smoking increases the dysfunction of Paneth cells.	- Autophagy and Paneth cell function.	[21,22,23]
IL23R	- R381Q - G149R - V362I	- It is associated with a protective effect against IBD in certain population groups, by limiting an excessive immune response.	- IL-23 signaling and cytokine regulation.	[24,25,26]
CARD9	- S12N - S12NΔ11	- S12N—increases the risk of IBD. - S12NΔ11—reduces the risk of IBD.	- NF-κB-related immune signaling.	[27,28,29]

**Table 2:** Biomarkers used to monitor IBD activity and nutritional interventions.

Biomarker	Clinical Application	Limitations	References
calprotectin (FC)	Assessment of mucositis activity, monitoring response to treatment, and ruling out active endoscopic inflammation.	No standardization between laboratories, threshold values differ between CD and UC.	[173,174]
CRP, ESR	Assessment of systemic inflammation.	Low specificity.	[176,177,178]
Ferritin	Assessment of iron reserves.	Acute phase protein—elevated in active inflammation, may mask iron deficiency.	[179,180,181]
Vitamin D (25-OH)	Vitamin D deficiency assessment.	Common deficiencies in IBD; seasonal fluctuations in concentrations.	[180,181]
Biomarkers microbiome (SCFA, tryptophan metabolites, secondary bile acids)	Exploratory assessment of microbiota modulation and response to dietary interventions.	Not validated for routine clinical use; lack of analytical standardization.	[64,185]

## Data Availability

No new data were created or analyzed in this study.
